# A Case of Mild Trichohepatoenteric Syndrome With New Variant Mutation in SKIV2L Gene: Case Report

**DOI:** 10.7759/cureus.19404

**Published:** 2021-11-09

**Authors:** Rawia F Albar, Mohammed S Alghamdi, Enad F Alsulimani, Ahmed M Almasrahi, Khalid A Alsalmi

**Affiliations:** 1 Pediatrics, King Abdulaziz Medical City, Jeddah, SAU; 2 College of Medicine, King Saud Bin Abdulaziz University for Health Sciences, Jeddah, SAU

**Keywords:** poor weight gain, failure to thrive, skiv2l gene, intractable diarrhea of infancy, trichohepatoenteric

## Abstract

Trichohepatoenteric syndrome (THES) is a rare autosomal recessive genetic disease characterized by severe early onset diarrhea, woolly and brittle hair, immunodeficiency, and liver disease. A mutation in either *SKIV2L* or *TTC37* genes can cause the disease. We report a case of a 41-month-old girl who suffered from intractable watery diarrhea, hair abnormality, dysmorphic features, and poor weight gain. The diagnosis was made through whole-exome sequencing analysis. The analysis detected a new variant mutation (*c.1201G* > *A*) p. (Glu401Lys) in the *SKIV2L* gene. She was admitted once for poor weight gain and nasogastric tube (NGT) feeding, with which the patient showed improvement. She was discharged to go home on hypoallergenic baby formulas and a regular diet with improved weight gain.

## Introduction

Trichohepatoenteric syndrome (THES), or sometimes called syndromic diarrhea, a rare cause of intractable diarrhea of infancy, is an autosomal recessive inherited genetic disease characterized by severe early onset diarrhea, failure to thrive, facial dysmorphic features, woolly and brittle hair, immunodeficiency, liver disease, congenital heart disease, and mental retardation [1,2]. The first case was described by Stankler et al. in 1982 [3]. In 1994, Girault et al. explained it as a clinical entity with common features like severe secretory diarrhea, dysmorphic features, brittle woolly hair, and immune system abnormalities [3,4]. Approximately, the reported prevalence among live births in Western Europe is one case out of every 300,000 to 400,000 people. Moreover, it does not appear that there is any relationship between ethnic origin and THES [5].

Studies have shown that mutations in tetratricopeptide repeat domain-containing protein 37 (TTC37) and the superkiller viralicidic activity 2 (SKIV2L) cause THES with an autosomal recessive pattern of inheritance. Superkiller complex protein is a part of the RNA exome, and it is encoded by both *SKIV2L* and *TTC37* genes [6]. The mechanism by which a defect in the mRNA degradation system leads to the symptoms associated with SD/THES remains unclear. Since its initial description, immunological defects such as low serum concentration of immunoglobulins and decreased or absent antibody responses to vaccination have been reported [2].

Most untreated cases of THES die at a very young age due to liver cirrhosis and infections. That is why early diagnosis and management are important for better outcomes [7]. Diagnosis of THES is based on detecting the signs and symptoms in a patient presenting with intractable diarrhea. Intestinal biopsies are not that useful in confirming the diagnosis of THES because there are no specific changes associated with THES. However, intestinal biopsies are used to rule out other causes of intractable diarrhea. Therefore, genetic testing is the only way to confirm the diagnosis [8].

In this manuscript, we are reporting a case of THES with a new variant mutation in the *SKIV2L* gene, which we did not find in the literature review.

## Case presentation

This is a case of 41-month-old infant girl who presented with weight loss and intractable diarrhea associated with oral feeding. She is a product of full-term pregnancy, delivered through spontaneous vaginal delivery, with a birth weight of 2 kg, and she did not require admission to the neonatal intensive care unit (NICU). The pregnancy was uneventful. Parents are first-degree cousins, and the patient has two older healthy siblings.

At the age of six months, she presented to another hospital afebrile with a loss of weight and had watery, non-bloody diarrhea, six to seven times per day. Both endoscopy and biopsy from the duodenum were normal according to the mother. The patient was initially misdiagnosed with cow milk protein allergy, so hypoallergenic formula was given but there was no improvement, then after two months, she was switched to amino acid-based infant formula 200 ml five times per day. In the beginning, there was an improvement, but with time, she stopped gaining weight again. At the age of 10 months, she came to the gastroenterology and genetics teams at our facility with chronic diarrhea, poor growth, and abnormal hair. Her weight and height were 5.30 kg (<3rd percentile) and 61 cm (<3rd percentile), respectively. The diagnosis of THES was confirmed by whole exons sequence (WES) analysis, which identified the homozygous variant (c.1201G > A) p. (Glu401Lys) in the *SKIV2L* gene. Upon literature review, we did not find the mentioned variant mutation in any previous literature (Table 1).

**Table 1:** Whole-exome sequencing. Whole-exome sequencing identified the homozygous variant c.1201G > A p. Glu401Lys in the *SKIV2L* gene. OMIM: Online Mendelian Inheritance in Man; MAF: minor allele frequency

Gene (isoform)	OMIM-P (Mood of Inheritance)	Variant	Zygosity	MAF gnomAD (%)	Literature pubmid	Classification
SKIV2L	614602 (AR)	*c.1201G* > *A* p.(Glu401Lys) chr6:31930352	Homogenous	0	-	Variant of Uncertain Significance

At 12 months of age, she was admitted for dehydration and nasogastric tube (NGT) feeding due to poor weight gain. Her body measurements upon admission were 5.64 kg (<3rd percentile) for the weight, and her height was 63 cm (<3rd percentile). Upon examination, she had some dysmorphic features such as a depressed nasal bridge, broad forehead, low set ears, and scanty dry hair. During her admission, she was having watery diarrhea with mucus two to three times a day. She was managed with intravenous fluid and the clinical nutritionist prepared a high-calorie formula. The nutrition therapy plan was to provide 180 ml of hypoallergenic baby formula (0.67 kcal/ml) every four hours orally, as much as she can tolerate, and if she did not complete her meal, give the rest through the NGT. This plan provided her with 192 ml/kg fluids and 135 kcal/kg/day energy. Her laboratory workup results were sodium 138 mmol/l, potassium 3 mmol/l, chloride 113 mmol/l, aspartate aminotransferase (AST) 40 units/l, alanine aminotransferase (ALT) 30 units/l, and gamma-glutamyl transferase (GGT) 32 units/l. Her immunoglobulins workup showed low immunoglobulin E <25 au/ml, and normal immunoglobulin G and A levels. She was improving and gained 100 grams in two days. On the seventh day of admission, she was able to tolerate oral feeding, so the patient was discharged on the same plan and to add 1 ml of medium-chain triglycerides oil every other day.

**Figure 1:**
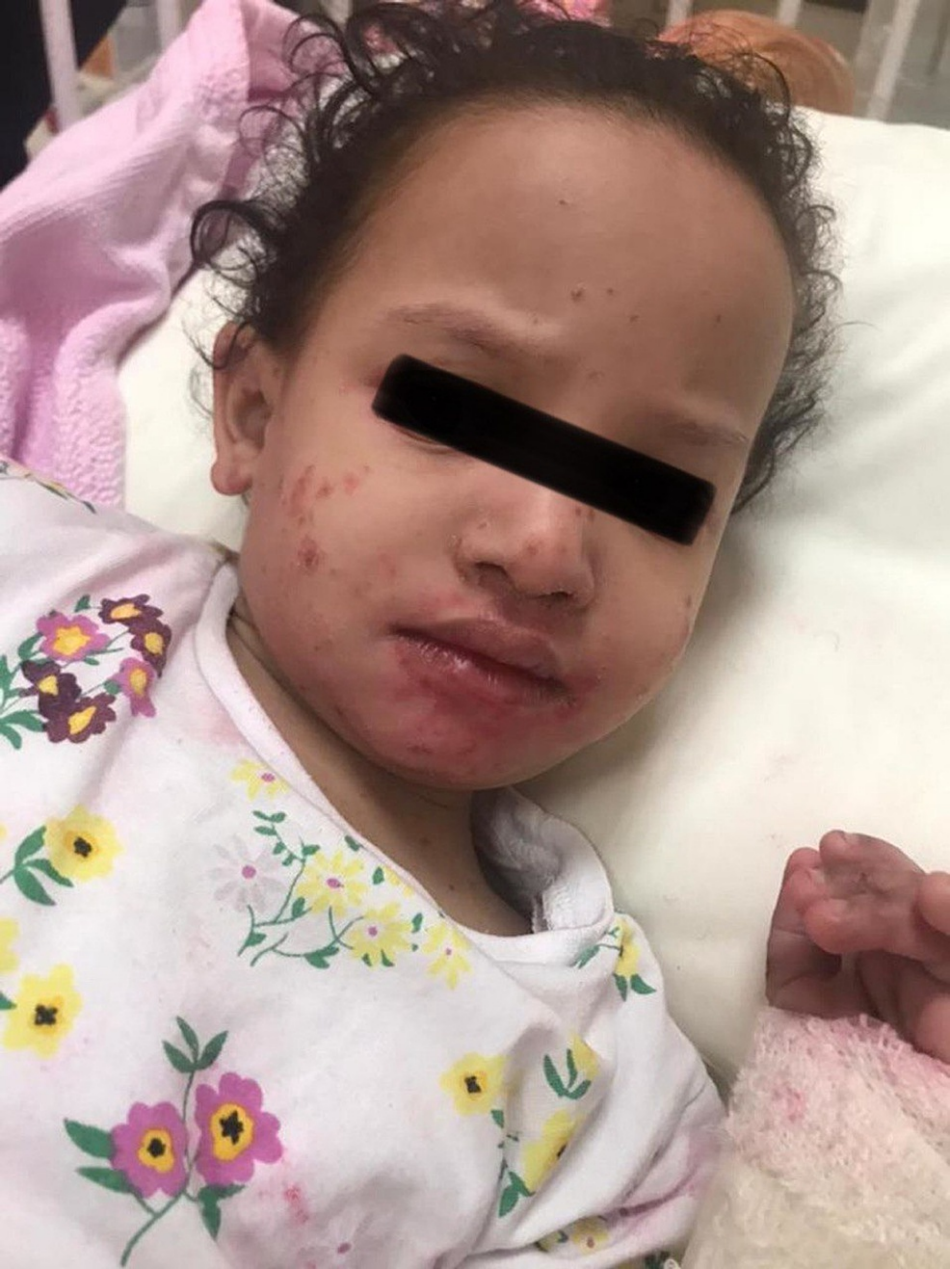
The patient at 41 months of age with bullous impetigo, showing dysmorphic facial features of THES. THES: trichohepatoenteric syndrome

At the age of 21 months, the mother reported in a follow-up visit that her daughter’s weight has been improved and reached 7.8 kg, but still under the third percentile. The patient was continued on hypoallergenic baby formulas and a regular diet. At the age of 41 months, the patient presented with progressive bullous itchy skin rash, and fluid-filled vesicles on erythematous background with red erosions and fissures all over the body but sparing abdomen and back. It was associated with cough, rhinorrhea, fever, and decreased urine output and oral intake, but without gastrointestinal complications. The diagnosis of bullous impetigo was made, and the patient was admitted. Amoxicillin, clavulanate, and diphenhydramine were administered intravenously with topical clindamycin and tretinoin cream. The patient showed clinical improvement in three days. The patient was discharged on oral Augmentin, Mupirocin, and Loratadine (Figure 1).

On follow-up evaluation at the age of 41 months, her weight and height were 10 kg (<3rd percentile) and 85 cm (<3rd percentile), respectively. She was able to tolerate normal oral feeding. She did not show any signs of intellectual disability, and she is developmentally up to her age now. She is active and plays with her cousins. She can speak and understand both Arabic and English, and according to the mother she is starting to memorize songs.

## Discussion

The main treatment of THES depends on total parenteral nutrition (TPN). Not all patients with THES can reach normal oral nutrition [9]. A literature review of 80 patients showed that 83% of them needed TPN treatment. Almost half of them weaned off TPN after 15 months. The remaining had to continue TPN [10]. Combined liver and small bowel transplantation are not a therapeutic option because of their complications [9].

A French cohort study with 15 cases of THES reported that all children showed typical THES symptoms, such as facial dysmorphism, intractable watery diarrhea, brittle and woolly hair, and immunodeficiency. Liver diseases and skin abnormalities were present in half of the patients. In addition, five of the cases passed away, mainly due to infections. According to the Kaplan-Meier method, the survival probabilities were 93.3%, 86.7%, 74.3%, and 61.9%, at 1 year, 5, 10, and 15 years of age, respectively. The percentage of patients who were small for gestational age was 80% of the total cases and 60% of the total patients had persistent short stature. Moreover, not only 60% of the cases had hemophagocytic disorder but also 60% of the patients suffered from mild mental retardation [11].

The onset of syndromic diarrhea symptoms was stated to be different from one study to another. A paper that was published in 2018 observed the onset of the disease in nine patients and noticed that the disease could begin within the first 335 days after delivery, with a birth weight range of 1 kg to 2.95 kg. Furthermore, facial dysmorphic features and skin abnormalities are very frequent symptoms with THES [12].

Our patient didn’t have any symptoms or complications at birth and didn’t need NICU admission. She started to have intractable diarrhea and poor weight gain at the age of 6 months. She could tolerate oral feeding most of her life. In contrast to most cases of THES, our patient was never on TPN throughout her life. The only time in which the patient needed NGT feeding was at the age of 12 months due to the need for rapid hydration and feeding when she presented with some signs of failure to thrive. The patient has some dysmorphic features such as a depressed nasal bridge, broad forehead, low set ears, and woolly hair. Immunodeficiency, heart disease, hepatic dysfunction, skin abnormality, or mental retardation weren’t detected.

In another case report, a 3-month-old patient complained of an inguinal hernia. However, after the surgery was done, the patient started to develop severe lower respiratory tract infection, pleural effusion, ascites, and liver cirrhosis [13]. Whereas in our case, the patient only complained of chronic diarrhea and did not get complicated by a serious infection or other diseases.

A recent paper that was done by Bourgeois et al. reports and analyzes the phenotypic and genotypic features of THES patients with a detailed literature review together with a cohort study. These studies included 96 cases that either carried a mutation in *SKIV2L* or a mutation in the *TTC37* gene. The goal was to compare the severity of the symptoms between the carriers of these mutations, the complications found in different cases, the exact location of the mutation, and finally their management plan. According to the paper, patients with *SKIV2L* mutation appear to be severely affected compared to patients with *TTC37* mutation, in regards to liver disease and prenatal growth impairment [14]. What was reported by Bourgeois et al. does not match with our case since our patient has a mutation in *SKIV2L* with mild symptoms and without liver damage or growth impairment.

## Conclusions

THES is a rare genetic disease, and it needs to be in the physician’s differential diagnosis of infants with intractable diarrhea, dysmorphic features, and abnormal hair. It needs a meticulous evaluation including endoscopy and intestinal biopsy to rule out the other causes. TPN is the main treatment, but NGT feeding and changing milk formula should be considered as our patient showed improvement with it.
